# 807. Optimizing Autologous Plasma Concentrations for Cultured Epidermal Autografts Cultivated Using a Modified Culture Technique

**DOI:** 10.1093/jbcr/irag033.185

**Published:** 2026-04-06

**Authors:** Wayne George Kleintjes, Tarryn Kay Prinsloo

**Affiliations:** Sheik Khalifa Hospital Fujairah, Cape Town, Western Cape; Cape Peninsula University of Technology, Cape Town, Western Cape

## Abstract

**Introduction:**

Cultured epidermal autografts (CEA) require platelet-rich plasma (PRP) to support cell growth and prevent desiccation during cultivation. Fresh PRP is typically applied daily over the two-week culture period; however, repeated blood withdrawal may be unfeasible in some patients. Furthermore, the optimal PRP concentration for CEA growth remains undefined. Identifying effective yet lower PRP concentrations may reduce patient burden while maintaining graft quality. We hypothesized that a lower PRP concentration could support CEA growth comparable to undiluted PRP.

**Methods:**

In this in vitro observational study, epidermal cells were enzymatically retrieved with trypsin (2-hour soak) from full-thickness skin biopsies, exposed to graded PRP dilutions (0–100%, v/v), and incubated in a pediatric incubator at 37°C for seven days. Histological assessments were performed on eosin-stained suspensions under light microscopy (4–100X). Macroscopic features, including cell density, gel formation, and fluid volume, were also evaluated.

**Results:**

PRP concentrations of 50% and 75% produced more viscous suspensions with higher cell-to-gel ratios and superior histoarchitecture compared with lower concentrations. These included keratinocyte encapsulation, epidermal stratification, dermal fiber development, and adnexal structure formation. Growth at these concentrations was comparable to that observed with undiluted (100%) PRP.

**Conclusions:**

These preliminary findings suggest that ≥50% PRP concentrations can support CEA growth patterns similar to 100%, indicating that reduced PRP volumes may be feasible without compromising culture quality. Further studies are needed to confirm optimal dosing protocols and explore whether low-volume strategies can minimize blood withdrawal requirements while maintaining therapeutic efficacy.

**Applicability of Research to Practice:**

The use of intermediate PRP concentrations (following validation) could significantly reduce the volume and frequency of blood draws required during CEA preparation. This approach may improve patient safety and comfort, particularly in pediatric or critically ill populations where blood conservation is essential. Clinically, establishing standardized, lower-volume PRP protocols could make CEA culture more accessible and sustainable, enhancing its adoption for both life-saving and cosmetic applications in diverse healthcare settings.

**Funding for the study:**

N/A.

